# Up-Regulated MISP Is Associated With Poor Prognosis and Immune Infiltration in Pancreatic Ductal Adenocarcinoma

**DOI:** 10.3389/fonc.2022.827051

**Published:** 2022-03-30

**Authors:** Xinyang Huang, Liangchao Zhao, Yixun Jin, Zhuoxin Wang, Tong Li, Hui Xu, Qi Wang, Lifu Wang

**Affiliations:** ^1^ Department of Gastroenterology, Ruijin Hospital, Shanghai Jiao Tong University School of Medicine, Shanghai, China; ^2^ Department of General Surgery, Ruijin Hospital North, Shanghai Jiao Tong University School of Medicine, Shanghai, China

**Keywords:** MISP, pancreatic ductal adenocarcinoma, prognosis, progression, immune infiltration, tumor mutational burden (TMB)

## Abstract

**Background:**

Pancreatic ductal adenocarcinoma (PDAC) is a highly malignant disease with a poor prognosis. More effective biomarkers and treatment options remain to be discovered. Mitotic Spindle Positioning (MISP), also called C19orf21, has been reported to be upregulated in several malignancies. However, the effects of MISP on PDAC have yet to be investigated.

**Materials and Methods:**

The differential expression of MISP at the mRNA and protein levels were evaluated using Gene Expression Profiling Interactive Analysis 2 (GEPIA 2), Gene Expression Omnibus (GEO), and the Human Protein Atlas (HPA) databases, and was further verified by quantitative real-time PCR and western blotting in PDAC cell lines. Correlations between MISP expression and clinical characteristics were explored using Kaplan-Meier Plotter Database and clinical data from The Cancer Genome Atlas (TCGA). CCK-8 assays, Transwell assays, and immunoblotting were used to determine the role of MISP in PDAC proliferation, migration, invasion, and epithelial-mesenchymal transition (EMT) *in vitro*. Gene Ontology (GO), Kyoto Encyclopedia of Genes and Genomes (KEGG) pathway analysis were executed by the R package ‘clusterProfiler’. Correlations between MISP expression and immune cell infiltration, immune checkpoints, immunophenoscore (IPS) and the tumor mutational burden (TMB) in PDAC were explored using the R package ‘CIBERSORT’, the Tumor Immune Estimation Resource 2.0 (TIMER2.0), and The Cancer Immunome Atlas (TCIA) database based on TCGA data.

**Result:**

MISP expression was significantly higher in pancreatic cancer tissues compared to normal pancreas tissues, which was associated with a poor prognosis. Increased expression of MISP was related to the proliferation, migration and invasion of PDAC cell lines. GO and KEGG pathway analyses determined that MISP is involved in the Ras signaling pathway and immune regulation. Higher expression of MISP was associated with decreased infiltration levels of activated CD4+ memory T cells, CD8+ T cells, M2 macrophages and neutrophils. Furthermore, increased MISP was associated with lower expression of immune checkpoint molecules, higher gene mutation burden and IPS.

**Conclusions:**

This study reveals that MISP, which is associated with the progression and prognosis of PDAC, may exert a potential regulatory effect on immune infiltration and predict the response to immunotherapy in PDAC.

## Introduction

Pancreatic ductal adenocarcinoma (PDAC) has become a common cause of cancer-related death according to 2021 Cancer Statistics (1). Certain mechanisms of PDAC remain unclear. Resistance to current treatment and late diagnosis contribute to almost identical incidence and mortality rates in patients with PDAC. Consequently, early detection and more efficient treatments are urgently needed.

Based on three microarray gene profiling datasets from the Gene Expression Omnibus (GEO) and RNA-Seq expression data from The Cancer Genome Atlas (TCGA), Mitotic Spindle Positioning (MISP or C19orf21), a substrate of Plk1, was found to be up-regulated in many types of cancer. It is involved in correcting mitotic spindle positioning and centrosome clustering (2, 3) and inducing stress fibers and other thick actin filaments (4). Furthermore, MISP has been reported to interact with and regulate IQ motif containing GTPase activating protein 1 (IQGAP1), a protein that plays a vital role in the progression of PDAC by facilitating epithelial-mesenchymal transition (EMT) and affecting the tumor microenvironment (5, 6). However, the expression and function of MISP in human pancreatic cancer are still unclear.

Supernumerary centrosomes are commonly observed in many human cancers (7, 8). Normal cells and cancer cells with supernumerary centrosomes universally undergo cell death due to the multipolar spindle-generated aneuploidy and prolonged arrest of checkpoints in the mitotic process (9, 10). However, in cancer cells, supernumerary centrosomes can cluster at opposite poles, thus forming a pseudobipolar structure to avoid lethal mitotic cataclysm (10, 11). EMT, originally described in embryonic development and tissue remodeling, is reported to be involved in the migration and invasion of cancer cells in PDAC (12, 13). Through EMT, cancer cells lose their epithelial characteristics and master the ability to disseminate. With downregulated expression level of E-cadherin and ZO-1, and upregulated expression levels of N-cadherin, Slug, vimentin and ZEB1, tumor cells obtain stronger capacity of migration and metastatic spread (14–16). All these events result in key molecular changes and are associated with PDAC therapy.

This study explored the role of MISP in PDAC through basic experiments in cell lines and integrating the data from TCGA, GEO, Human Protein Atlas (HPA), Gene Expression Profiling Interactive Analysis 2 (GEPIA 2), Kaplan-Meier Plotter, Gene Ontology (GO), Kyoto Encyclopedia of Genes and Genomes (KEGG), Tumor Immune Estimation Resource 2.0 (TIMER2.0), and The Cancer Immunome Atlas (TCIA) through bioinformatics analysis. The flowchart of our work is shown in **Supplementary Figure 1**.

## Materials and Methods

### GEO Data Acquisition and Analysis

Series matrix and probe annotation of three PDAC datasets (GSE62452, GSE32676 and GSE71729) were obtained from GEO database (https://www.ncbi.nlm.nih.gov/geo/). We transformed the probe ID into gene symbols. If multiple probe sets matched the same gene, the average expression value would be calculated as the gene expression value. The expression of MISP(C19orf21) was compared in the normal group and the PDAC group using an unpaired t-test. Statistical analysis was conducted using GraphPad Prism version 8.

### TCGA Data Acquisition and Analysis

TCGA-pancreatic adenocarcinoma (PAAD) RNA-Seq expression data (FPKM format) and the corresponding patient clinical information were obtained from TCGA database (https://portal.gdc.cancer.gov/), which comprised 172 PAAD samples (171 patients) and excluded cystic, mucinous, serous neoplasms and samples not otherwise specified. All samples were utilized to explore differences in MISP expression using an unpaired t test or a nonparametric test. Box plots were used to visualize the relationships between MISP expression and clinical characteristics: stage and grade of cancer, age, gender, or history of alcohol use, smoking, diabetes, chronic pancreatitis (CP), tumor size, radiation therapy history, and anatomic neoplasm subdivision. Gene expression data and clinical data were stored for further bioinformatics analysis.

### GEPIA 2 Database Analysis

The GEPIA 2 database (http://gepia2.cancer-pku.cn/#index), which combines gene expression data from TCGA and GTEx, was exploited to explore the expression of MISP in PDAC and other cancer types in this study.

### Kaplan-Meier Plotter Database Analysis

Kaplan-Meier Plotter (http://kmplot.com/analysis/) is a publicly-available comprehensive database that evaluates the relationship between gene expression and the survival trend of multiple cancers (17). The prognostic data of PAAD in this database is totally based on TCGA-PAAD dataset. This database was used to investigate the correlation between MISP expression and the prognosis of PDAC.

### Human Protein Atlas Database Analysis

Human Protein Atlas (HPA) (https://www.proteinatlas.org/), a public database, was applied to assess protein expression profiles in human tumor tissues. The levels of MISP protein expression in normal pancreas and PAAD tissue were explored in this database.

### Cell Culture and Reagents

Human pancreatic cancer cell lines (BxPC-3, PANC-1, AsPC-1, and Capan-1) were obtained from the American Type Culture Collection (ATCC). MIA PaCa-2, SW1990, and the normal human pancreatic ductal cell line hTERT-HPNE were purchased from the Cell Bank of the Chinese Academy of Sciences. All cells were cultured in Dulbecco’s modified Eagle medium (DMEM: Gibco; Hyclone; Meilunbio) containing 10% fetal bovine serum (FBS: Gibco) and 1% penicillin/streptomycin (Meilunbio) at 37°C in an atmosphere with 5% CO2.

### Quantitative Real-Time PCR and Western Blotting

For qRT-PCR, total RNA was isolated with TRIzol reagent (Invitrogen), and cDNA was synthesized using the First Strand cDNA Synthesis kit (Yeasen). For the quantitative real-time PCR (qRT-PCR), the SYBR Green Supermix kit (Yeasen) was used on the StepOnePlus™ Real-Time PCR System (Applied Biosystems). The primer sequences for qRT-PCR were as follows:

MISP forward: CCCTGAGCACAAAGCAAGAG,
MISP reverse: GCAGATCAGATGACTGGGACTT;
GAPDH forward: GGAGCGAGATCCCTCCAAAAT,
GAPDH reverse: GGCTGTTGTCATACTTCTCATGG.

Western blotting procedures were described in our previous study (18). The primary antibodies used were MISP (1:1,000, cat.no. 26338-1-AP, Proteintech); ZO-1 (1:1,000, cat. no. 8193P; Cell Signaling Technology); ZEB1 (1:1,000, cat. no. 70512S; Cell Signaling Technology), N-cadherin (1:1,000, cat. no. 13116T; Cell Signaling Technology); vimentin (1:1,000, cat. no. 3932S; Cell Signaling Technology); E-cadherin (1:1,000, cat.no. 20874-1-AP, Proteintech.), Slug (1:1,000, cat. no. 9585T; Cell Signaling Technology, Inc.) and β-actin (1:2,000, cat.no.GB12001, Servicebio.), GAPDH (1:2,000, cat.no. GB12002, Servicebio.); HRP-linked secondary antibodies (1:5,000; cat. no. 7074P2 and 7076S; Cell Signaling Technology, Inc.).

### Lentivirus-Mediated Overexpression

MISP was overexpressed using PGMLV-CMV-H_MISP-EF1-ZsGreen-T2A-PURO (Genomeditech Biotechnology). Cells were cultured to a density of 50% in 6-well plates according to the manufacturer’s protocol. Stable transfected cells were selected by culture medium with 2-4 μg/mL puromycin.

### CCK-8 Assay

Transfected MIA PaCa-2 cells (1×10^3^ cells/well) and SW1990 cells (1.5×10^3^ cells/well) were seeded in 96-well plates. 100 μL DMEM containing 10 μL CCK-8 reagent (Meilunbio, Han Bio) was added to each well at 5 distinct time points. After incubation in the dark at 37°C for 2 h, absorption was detected at the 450 nm wavelength. Each group consisted of five duplicate wells, and the experiments were repeated in triplicate.

### Transwell Migration and Invasion Assays

Transwell migration assay was performed using Transwell chambers (24-well, 8 μm pore size; Corning or Jet Bio-Filtration). The invasion assay was conducted using chambers coated with BD matrigel matrix (BD Bioscience). Approximately 5–15×10^4^ cells were seeded in the upper chambers with DMEM, and 800μl DMEM containing 10% FBS was added to the lower chambers. Plates were incubated at 37°C for 24-48 hours. Cells that did not migrate or invade through the pores were wiped using wet swabs, those on the lower side of the filter were fixed with 4% paraformaldehyde, stained with crystal violet, and finally counted to evaluate the migratory and invasive abilities.

### Tumor Mutational Burden Analysis

Using the R package “Maftools”, we visualized mutation profiles of PDAC with high- and low-MISP expression. The tumor mutational burden (TMB) was calculated by dividing the total variant count (base substitutions, insertions, deletions, or insertions across bases) by the total exon length. Then the difference in TMB between the MISP high and low expression subgroups was detected using the Wilcoxon test.


$$TMB=\frac{Total\;variant\;count}{Total\;exon\;length}$$

### Analysis of Differentially Expressed Genes and Co-Expressed Genes

The RNA-seq FPKM data relative to PAAD obtained from TCGA (https://portal.gdc.cancer.gov/; n=171) were used to screen differentially expressed genes (DEGs) between the high- and low- MISP subgroups, which were defined by the median expression level of MISP. DEGs were screened using the Wilcoxon test. Significant DEGs were defined based on the following cutoff values: False discovery rate (FDR)<0.05, |log fold change (FC)| >1. Co-expressed genes were screened out through Pearson’s correlation analysis. Genes with P <0.01 and R>0 were defined as positively co-expressed genes, while genes with P <0.01 and R<0 were defined as negatively co-expressed genes.

### GO and KEGG Pathway Analysis

GO enrichment was applied to analyze the biological function of certain genes from three different aspects: biological processes, molecular functions, and cellular components. KEGG analysis was used to search biological pathways containing enriched genes. The R package ‘clusterProfiler’ was applied to conduct the GO and KEGG pathway analyses based on DEGs and co-expressed genes.

### Immune-Relevant Genes and Immune Infiltration Analysis

The list of immunologically relevant genes (IRGs) was downloaded from the ImmPort Portal database (https://www.immport.org/home/) (19). The correlation between MISP and immune infiltration was explored using two different tools according to TCGA_PAAD cohort. First, the proportions of 22 kinds of immune cells in the 171 patients were determined using the R package ‘CIBERSORT’ (20). Samples with an output of P-value<0.05 indicated the inferred fractions of immune cell populations produced by CIBERSORT were statistically significant. Differences in immune cell fractions between high- and low-MISP groups was then analyzed by Wilcoxon test. The specific type of immune cells correlated with the prognosis of patients with PDAC were analyzed by Kaplan-Meier analysis. Furthermore, the correlation between MISP (C19orf21) expression and the level of immune cell infiltration in PAAD was evaluated in the Tumor Immune Estimation Resource 2.0 (TIMER2.0) database (http://timer.cistrome.org/) (21), an open-source repository of immunity and gene expression.

### Correlation Between MISP and Immune Checkpoints and Immunophenoscore (IPS) Analysis

The RNA-seq FPKM data of PAAD from TCGA were used to evaluate the expression of immune checkpoint molecules. Differences in the expression of immune checkpoints (ICs) between the high- and low-expression MISP subgroups were analyzed by the Wilcoxon test. The correlation between ICs and MISP was calculated using Pearson’s correlation analysis. The IPS calculation process was performed as described in a previous article (22). Higher scores represented a better outcome with ICI treatment. IPS can be obtained from TCIA site (https://tcia.at/home).

## Results

### The Expression of MISP Increased in PDAC Tissues

**Figure 1:**
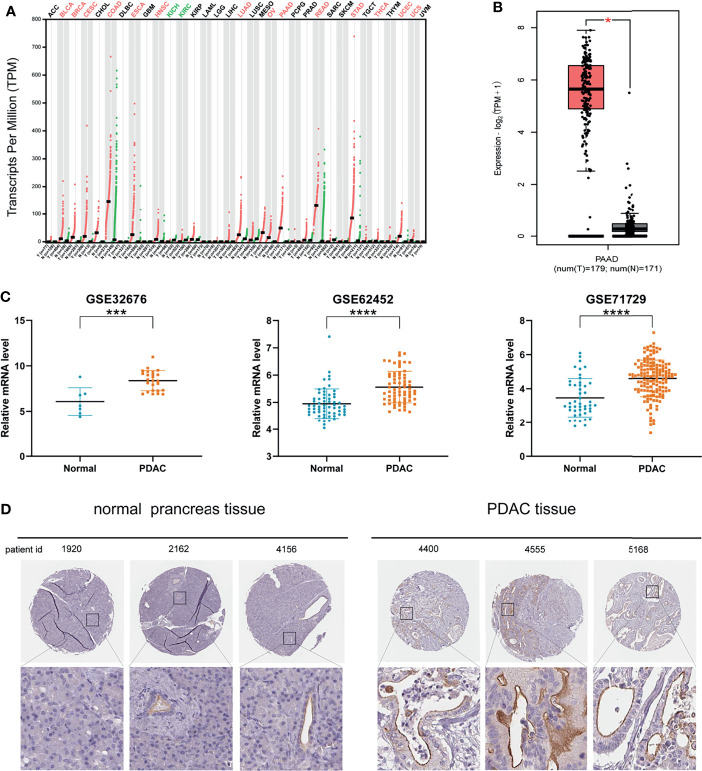
MISP expression is increased in PDAC tissues based on GEPIA 2 and GEO datasets. **(A)** MISP was significantly upregulated in various tumors. **(B)** Expression level of MISP in PAAD in comparison with the normal group as analyzed by the GEPIA 2 database. **(C)** The GEO datasets (GSE32676, GSE62453 and GSE71729) showed that the MISP mRNA expression is upregulated in PDAC tissue. **(D)** Representative immunohistochemistry staining images for MISP in PDAC and normal tissue from the HPA database. *P<0.05, ***P<0.001, ****P<0.0001.

The results analyzed by GEPIA 2 database showed that MISP was up-regulated in bladder urothelial carcinoma (BLCA), breast invasive carcinoma (BRCA), cervical squamous cell carcinoma and endocervical adenocarcinoma (CESC), colon adenocarcinoma (COAD), esophageal carcinoma (ESCA), head and neck squamous cell carcinoma (HNSC), lung adenocarcinoma (LUAD), ovarian serous cystadenocarcinoma (OV), PAAD, rectum adenocarcinoma (READ), stomach adenocarcinoma (STAD), thyroid carcinoma (THCA), uterine corpus endometrial carcinoma (UCEC), and uterine carcinosarcoma (UCS). In contrast, MISP was down-regulated in kidney chromophobe (KICH) and kidney renal clear cell carcinoma (KIRC) **(** **Figure 1A** **)**. Gene expression data from TCGA were analyzed by GEPIA 2 and three datasets (GSE32676, GSE62452, GSE71729) were obtained from the GEO database, which revealed the up-regulation of MISP in PDAC tissue than in non-cancerous pancreatic tissues **(** **Figures 1B, C** **)**. Furthermore, we analyzed immunohistochemical samples from HPA, which confirmed the level of MISP expression was higher in PDAC tissue than in normal samples **(** **Figure 1D** **)**.

### MISP Accumulated During Clinical Progression and Influenced the Prognosis of PDAC

**Figure 2:**
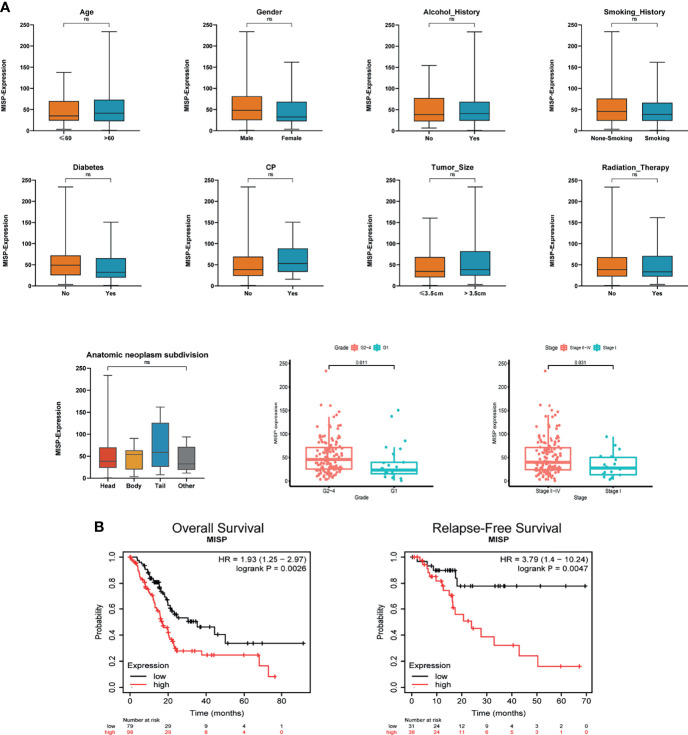
Clinical correlation and prognosis analysis based on TCGA and Kaplan-Meier Plotter database. **(A)** Clinical correlation based on TCGA **(B)** OS and RFS analysis in the Kaplan-Meier Plotter database based on TCGA data. ns, not significant.

The clinical data of the PDAC patients was obtained from TCGA database. In clinicopathological assessment, pronounced differences were observed in MISP expression across different pathological processes, including grade and stage. Increased expression was observed in Grades 2 to 4 and in Stages II to IV than in Grade 1 and Stage I (P<0.05). However, there was no evidence indicating that MISP expression was associated with age, gender, history of alcohol use, smoking, diabetes, or CP, tumor size, radiation therapy, or anatomic neoplasm subdivision **(** **Figure 2A** **)**. The prognostic analysis in Kaplan-Meier Plotter database indicated that high expression of MISP reduced the overall survival (OS) and relapse-free survival (RFS) of patients with PDAC **(** **Figure 2B** **)**.

### MISP Promoted the Cell Proliferation

**Figure 3:**
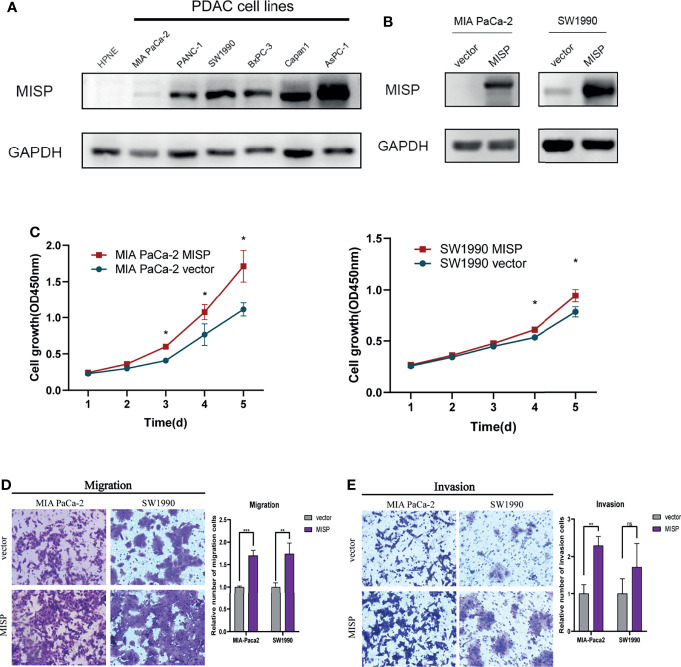
The effects of MISP expression on PDAC cell proliferation, migration, and invasion. **(A)** MISP protein expression levels were detected in hTERT-HPNE and several PDAC cell lines. GAPDH was used as the loading control. **(B)** The overexpression efficiency of MISP was measured by western blotting. **(C)** CCK-8 assays demonstrated that the overexpression of MISP promoted the proliferation of MIA PaCa-2 and SW1990. *P<0.05. **(D, E)** Transwell migration and invasion assays. MISP-overexpressed MIA PaCa-2 and SW1990 exhibits significantly increased migration and invasion capacity than the negative controlled infectants, exclusive of the invasion capacity of SW1990 cells. *P < 0.05; **P < 0.01; ***P < 0.001; ns, not significant.

To further support our hypothesis that MISP accumulation in advanced-stages impacted on the prognosis of PDAC patient, *in-vitro* experiments were performed as follows. We evaluated the expression of MISP in normal pancreatic epithelial cells (hTERT-HPNE) and 6 PDAC cell lines (MIA PaCa-2, PANC-1, SW1990, Capan1, BxPC-3, and AsPC-1) by qRT-qPCR **(** **Supplementary Figure 2** **)** and western blotting **(** **Figure 3A** **)**. MISP expression was strikingly increased in PDAC cell lines in contrast to the normal pancreatic cell line. We then stably overexpressed MISP in MIA PaCa-2 and SW1990 cells by lentivirus-delivery and confirmed the transfection efficiency by western blotting **(** **Figure 3B** **)**. Using these cell lines, we showed that cell proliferation was potentiated by the overexpression of MISP *via* CCK-8 assay (P<0.05) **(** **Figure 3C** **)**.

### MISP Propelled the Migration and Invasion in PDAC Cells

We evaluated the effect of MISP on migration and invasion of PDAC cells *in vitro*. The results showed that the overexpression of MISP markedly promoted migration and invasion in MIA PaCa-2 cells, and migration ability in SW1990 cells **(** **Figures 3D, E** **)**.

### Patients’ Characteristics

We divided 171 patients from TCGA database into two groups according to the level of MISP expression; the clinical factors for PDAC did not appear to differ between these two subgroups **(** **Table 1** **)**. The high- and low-expression MISP subgroups were used to explore TMB, the DEGs, pathways enrichment, and immune infiltration in the following analysis.

**Table 1:** Baseline characteristics of PDAC patients stratified by MISP expression.

Clinical parameters	Overall (n=171)	MISP mRNA expression		P value
		Low(n=86)	High(n=85)	
**Gender**				
Male	93	41	52	0.092*
Female	78	45	33	
**Age**				
Mean ± SD	64.76 ± 10.82	63.186 ± 9.672	66.3529 ± 11.713	0.062**
**Grade**				
G1	30	20	10	0.112***
G2	92	45	47	
G3	47	20	27	
G4	1	1	0	
GX	1	0	1	
**Stage**				
Stage I	20	13	7	0.603***
Stage II	141	67	74	
Stage III	3	2	1	
Stage IV	4	2	2	
Stage X	3	2	1	
**T**				
T1	6	3	3	0.681***
T2	23	15	8	
T3	137	65	72	
T4	3	2	1	
TX	2	1	1	
**M**				
M0	75	44	31	0.151***
M1	4	2	2	
MX	92	40	52	
**N**				
N0	47	26	21	0.380***
N1	120	57	63	
NX	4	3	1	

*Pearson chi-squared test.

**Independent sample t-test.

***Fisher exact test.

### Correlation Between MISP Expression and the Mutational Status

**Figure 4:**
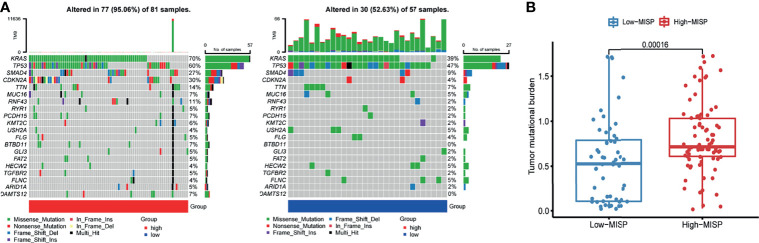
Mutation profile and TMB of PAAD samples from TCGA database. **(A)** Waterfall plot of mutation profiles of each gene in each sample in high- and low-MISP group. The legend at the bottom described the mutation types. The plot above the legends showed the mutation burden of each sample. **(B)** TMB in high- and low- MISP group.

The mutation frequencies of KRAS, TP53, SMAD4, CDKN2A, TTN, and MUC16 were the highest in both high- and low-MISP subgroups. Nevertheless, the percentage of samples harboring gene mutations was markedly higher in patients with higher MISP expression (95.06%) than in patients with weaker MISP expression (52.63%) **(** **Figure 4A** **).** Higher TMB was identified in patients with higher expression of MISP than in those with weak MISP expression (P<0.001) **(** **Figure 4B**).

### KEGG and GO Analysis of DEGs and Co-Expressed Genes

**Figure 5:**
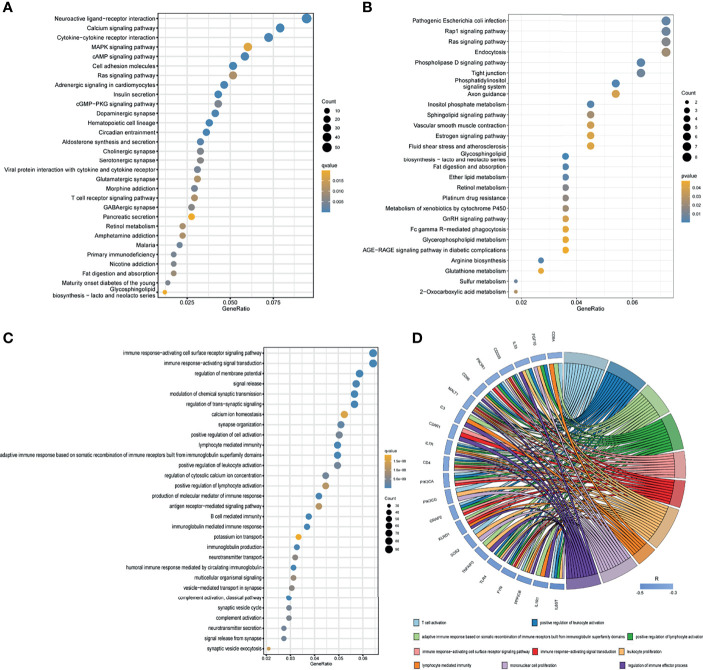
Results of KEGG enrichment and GO analysis. **(A)** KEGG pathway analysis of DEGs based on TCGA database. **(B)** KEGG pathway analysis of co-expressed genes based on TCGA database. Only pathways with a P-values <0.05 are presented. **(C)** GO analysis (biological process) of DEGs based on TCGA database. **(D)** GO analysis (biological process) of co-expressed immune genes in PDAC. Negatively co-expressed IRGs are shown.

Through differential expression gene analysis, 2292 DEGs (|logFC|> 1, FDR<0.05) were identified based on RNA-seq data from TCGA_PAAD cohorts. Similarly, MISP co-expressed genes were screened from TCGA_PAAD datasets, and 289 genes having a strong correlation with MISP expression were identified (|R|>0.5, P<0.01). These genes were then applied to the KEGG pathway analysis **(** **Figures 5A, B** **)**, which revealed that DEGs and co-expressed genes were both enriched in the RAS signaling pathway.

In the GO analysis of DEGs, we discovered certain DEGs were enriched in immune-related pathways **(** **Figure 5C** and **Supplementary Figure 3** **)**, which prompted us to seek the intersection between co-expressed genes and IRGs. A total of 613 MISP co-expressed IRGs (P<0.01) were identified for subsequent GO analysis **(** **Supplementary Figure 4** **)**. The biological processes which the most negatively co-expressed IRGs enriched in are shown in **Figure 5D**.

### MISP Induced EMT in PDAC Cells

**Figure 6:**
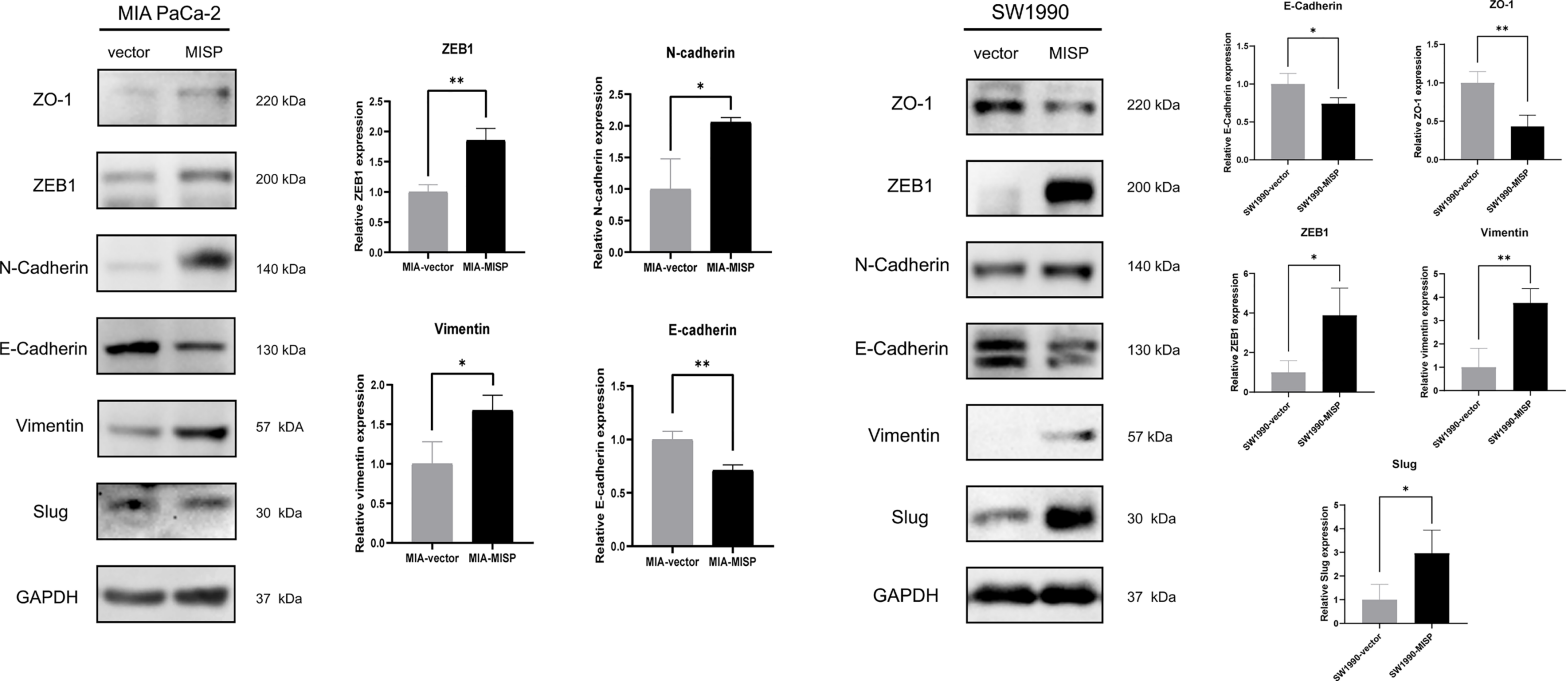
MISP induces EMT in PDAC cells. The protein expression of EMT markers (ZO-1, N-cadherin, E-cadherin, and vimentin) and EMT-TFs (Slug, ZEB1) were measured by western blotting. GAPDH was used as the loading control. MIA: MIA PaCa-2 cells. *P<0.05, **P<0.01.

EMT, widely known as a crucial factor in tumor metastasis, allows cancer cells to become more invasive and develop metastatic growth characteristics. Inspired by the results of Transwell assays and pathway enrichment analyses above, we evaluated the influence of MISP during the EMT process by measuring the expression of EMT markers and EMT-inducing transcription factors (EMT-TFs) in the treated MIA PaCa-2 and SW1990 cell lines. E-cadherin had lower expression while N-cadherin, vimentin, and ZEB1 had higher expression in the MISP-overexpressed MIA PaCa-2 cells, but there were no significant changes in Slug and ZO-1 between the MISP overexpressed cells and the negative controlled group. ZO-1 and E-cadherin had lower expression while vimentin, Slug and ZEB1 had higher expression in the MISP-overexpressed SW1990 cells, but there were no significant changes in N-cadherin in negative controlled SW1990 cells **(** **Figure 6** **)**.

### Bioinformatics Analysis of MISP-Modulation of Immune Infiltration and Response to Immunotherapy

**Figure 7:**
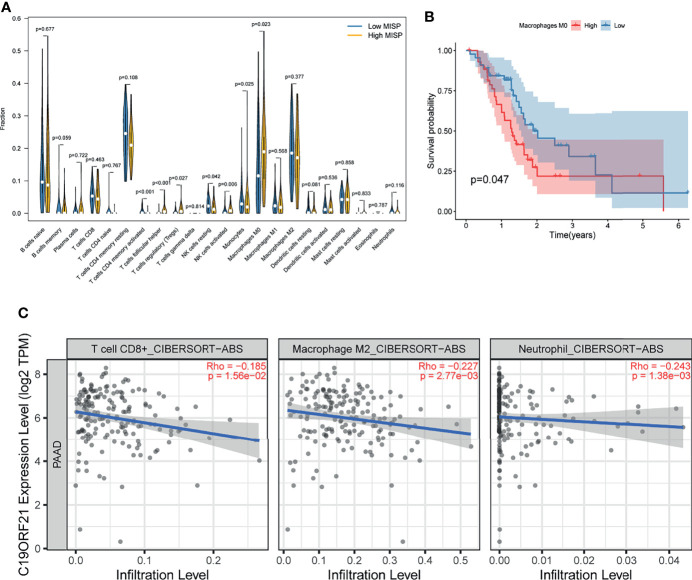
Immune infiltration result. **(A)** Comparison of immune infiltration between high- and low- MISP subgroups. **(B)** Relationship between infiltration of M0 macrophage and survival rate in PDAC patients as analyzed by Kaplan-Meier. **(C)** Correlation analysis between the expression level of MISP and immune infiltration (P<0.05).

The result of GO analysis of the DEGs and co-expressed genes prompted us to explore the correlation between MISP expression and immune infiltration. According to the analysis of the tumor-infiltrating immunocyte profiles through CIBERSORT algorithm based on TCGA database, we determined the degree of infiltration of 22 types of immune cells and obtained data relative to 96 patients with significant results (P<0.05). The infiltration levels of follicular helper T cells (Tfh), regulatory T cells (Tregs), activated natural killer (NK) cells, and M0 macrophages were higher in the high-MISP group, while activated CD4+ memory T cells, resting NK cells, and monocytes showed the opposite result (P<0.05) **(** **Figure 7A** **)**. Survival analysis, conducted *via* the Kaplan-Meier survival method, showed that a high level of M0 macrophage infiltration predicted a poorer prognosis (P<0.05) **(** **Figure 7B** **).** Moreover, we analyzed the correlation between immune cell infiltration and MISP expression in the TIMER 2.0 using the Cibersort-ABS algorithm. The results demonstrated that the infiltration of CD8+ T cells, M2 macrophages, and neutrophil was negatively correlated with MISP expression **(** **Figure 7C** **)**, while other immune cell types showed no statistical correlation **(** **Supplementary Figure 5**).

**Figure 8:**
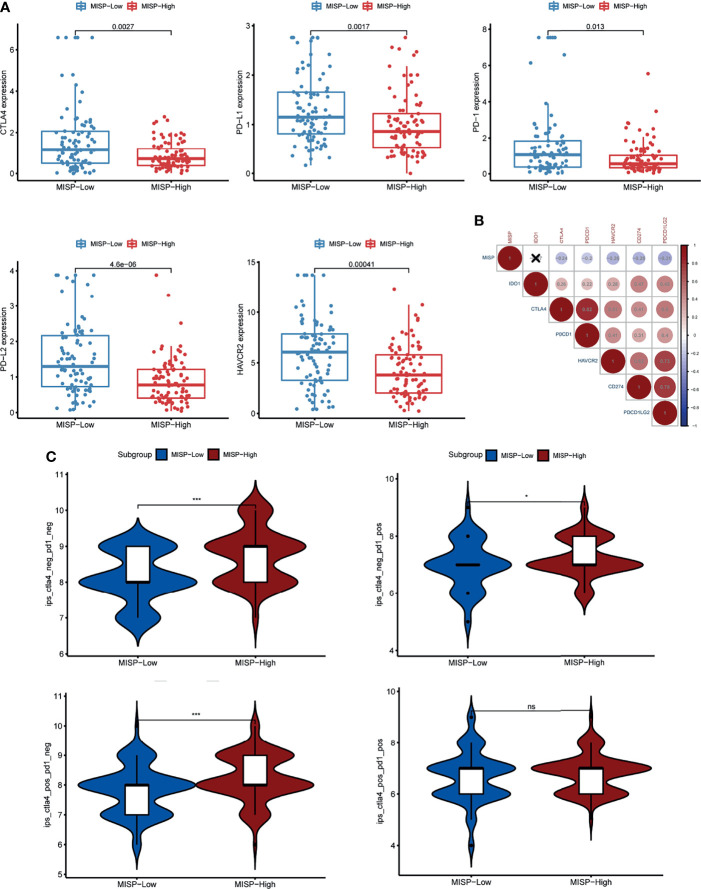
The association between MISP expression and response to immune checkpoint inhibitors (ICI). **(A)** The gene expression of PD-1, PD-L1, CTLA-4, PD-L2 and HAVCR2 in the high-MISP and low-MISP groups. **(B)** Pearson’s correlation test was used to explore the correlation of paired IC genes and MISP. **(C)** The difference of IPS between the high- and low-MISP subgroups. *P<0.05; ***P<0.001; ns, not significant.

The expression of six immune checkpoints (CTLA4, PD1, PD-L1, PD-L2, HAVCR2, IDO1) was analyzed between the MISP high- and low-expression groups. The results indicated that patients with lower MISP presented higher expression of immune checkpoint genes **(** **Figure 8A** and **Supplementary Figure 6** **).** Meanwhile, there were negative correlations between MISP expression and all other immune checkpoint molecules evaluated, except IDO1 **(**P<0.05) **(** **Figure 8B**). The IPS file downloaded from TCIA was used to evaluate whether MISP expression could predict the response to immunotherapy in PDAC patients. The IPS was obviously higher in the high-MISP group, which indicated patients with higher MISP expression would achieve a better response to immunotherapy **(** **Figure 8C** **)**.

## Discussion

Pancreatic adenocarcinoma is one of the most lethal tumors with a 5-year survival rate of about 9% (23). Albeit with a grim prognosis, conventional therapy remains the treatment of choice. (24, 25). Therefore, further exploration of the underlying carcinogenic mechanisms and the search for novel molecules as effective therapeutic targets remains imperative.

Bioinformatics analysis is widely used to identify effective cancer therapeutic and diagnostic targets. By analyzing data from public databases, MISP was identified as an up-regulated gene both at the mRNA and protein levels in PDAC tissue. The expression of MISP in PDAC cell lines was consistent with the online database findings. Clinical pathological characteristics, such as clinical stage and grade, were associated with MISP expression according to the TCGA_PAAD cohort. In addition, high expression of MISP was indicative of short OS and RFS. As a candidate oncogene in PDAC, MISP contributed to the proliferation, migration, and invasion of MIA-PaCa2 and SW1990 cell lines *in vitro*.

Certain mutated genes, such as KRAS and TP53, are common in PDAC and likely to influence the vital mechanism of tumorigenesis and tumor progression (26). KRAS, TP53, SMAD4, CDKN2A, TTN, and MUC16 were most frequently mutated in both high- and low-MISP expression group according to our analysis. However, obviously higher TMB was detected in MISP high-expression subgroup.

Inspired by these results, KEGG enrichment analysis was applied to the DEGs and co-expressed genes to define the potential functions of MISP in PDAC (**Figures 5A, B** **)**. Our findings suggested that the calcium signaling pathway, MAPK signaling pathway, cAMP signaling pathway, cell adhesion molecules, tight junction, cGMP-PKG signaling pathway, Rap1 signaling pathway and Ras signaling pathway were strongly correlated with MISP expression. Among these, the Ras signaling pathway was common to both DEGs and co-expressed genes. Furthermore, high expression of MISP was associated with greater KRAS mutational burden (**Figure 4A**). ERK Phosphorylation induced by mutated KRAS overactivates the RAS-MAPK signaling pathway, which may significantly contribute to pancreatic cancer progression (27).

Depletion of MISP has been proven to inhibit the directed migration of the UPCI : SCC114 cells, an oral squamous cancer cell line (2). According to our results, overexpressed MISP facilitated the process of migration and invasion in PDAC cells. EMT affects the capacity of migration and invasion, which may partly explain these processes (28, 29). In addition, the result of KEGG and GO analysis showed that the DEGs enriched in cell adhesion molecules and tight junction process, which could influence the process of EMT (30, 31). Herein, we found that MISP was associated with the downregulation of EMT-related protein E-cadherin, ZO-1 and upregulation of N-cadherin, slug vimentin and ZEB1, thus promoting the EMT process and the capacity for cell migration.

MISP is involved in centrosome clustering, interaction with focal adhesion kinase (FAK), and the induction of stress fibers (4). Centrosome clustering is essential for division and survival of cancer cells (32). To avoid lethal mitosis, cancer cells with amplified centrosomes achieve pseudo-bipolar division *via* clustering supernumerary centrosomes (33–36). Inhibiting supernumerary centrosome clustering in mitosis to induce defectively multipolar divisions and cell death has been proposed as a potential treatment for targeting tumor cells with high incidences of supernumerary centrosome, thus sparing normal cells (37, 38).Stress fibers may play a role both in cell migration and regulating nuclear function (39). FAK was shown to be involved in the progression of carcinogenesis including EMT and metabolism of malignant cells (40, 41). MISP depletion increases accumulation of IQGAP1 at the cell cortex and causes active Cdc42 reduction, which can be rescued by the overexpression of IQGAP1 (5). Activated Cdc42 can prolong the length of pseudopodia, remodel the actin cytoskeleton, and regulate epithelial cell polarization, which enhances cell migration, invasion and promotes PDAC progression (42–44). These results implied that MISP plays a critical role in the progression of PDAC through these pathways.

In this study, we also investigated the correlation between MISP expression and immune cell infiltration. The infiltration of Tfh, Tregs, activated NK cells, and M0 macrophages was significantly increased in high-MISP tumor tissues, while the infiltration of activated CD4+ memory T cells, CD8+ T cells, resting NK cells, monocyte, M2 macrophages and neutrophils was decreased. Kaplan-Meier survival analysis showed that the infiltration of M0 macrophages was associated with the prognosis of PDAC patients. GO analysis showed that IRGs negatively correlated with MISP expression were involved in leukocyte activation and proliferation, as well as immune response-activating biological processes. To survive, tumors evade the surveillance of immune system in the tumor microenvironment by manipulating immune cells (45, 46). MISP is likely to play a specific role in the limitation and suppression of T cell activity. Emerging evidence indicates that IQGAP1, FAK and mutated KRAS are implicated in the immunoregulation of tumors (47–49). Recent studies have suggested that Treg cells are linked to tumor immune surveillance and carcinogenesis (48, 50).

Our findings also indicated that the levels of five IC genes were lower in PDAC tissue with a high expression of MISP. However, higher TMB, which has been linked to high response rates to ICIs (51, 52), was identified in PDAC tissue with a high expression of MISP. IPS data, downloaded from the TCIA, provides a predictive score for evaluating a patient’s response to immune therapy, which has been confirmed in melanoma patients (22, 53). IPS was higher in the MISP-high expression group, indicating that individuals with high MISP expression may achieve a better response to ICI therapy. This study unveils that MISP, which has not yet been studied in PDAC, may be strongly correlated to the immune infiltration of PDAC, highlighting the potential value of MISP in determining response to immunotherapy.

In summary, we characterize MISP as an oncogenic factor that can potentially act as a prognostic predictor and therapeutic target for PDAC. Furthermore, our findings provide evidence supporting the significant role of MISP in promoting PDAC cell proliferation and influencing the EMT process, and its correlation with immune infiltration and TMB. Further *in-vitro* and *in-vivo* experiments are needed in the subsequent studies to clarify the underlying mechanism through which MISP promotes the progression and influences the immune infiltration of PDAC.

## Data Availability Statement

All datasets used in this study were available in the public databases which have been mentioned in *Materials and Methods* section. Further inquiries can be directed to the corresponding authors.

## Author Contributions

XH and YJ contributed to data collection, statistical analysis, results integration, and experiment performance. XH and LZ wrote and revised the manuscript and R scripts. LZ, ZW, TL, and HX executed data acquisition and statistical analysis. QW and LW designed and supervised the study, interpreted the data, and revised the manuscript. All authors contributed to the article and approved the submitted version.

## Funding

This work was supported by National Natural Science Foundation of China (Grant Nos. 81870385, 81672719, 81800419 and 81702740).

## Conflict of Interest

The authors declare that the research was conducted in the absence of any commercial or financial relationships that could be construed as a potential conflict of interest.

## Publisher’s Note

All claims expressed in this article are solely those of the authors and do not necessarily represent those of their affiliated organizations, or those of the publisher, the editors and the reviewers. Any product that may be evaluated in this article, or claim that may be made by its manufacturer, is not guaranteed or endorsed by the publisher.

## Abbreviations

PDAC, pancreatic ductal adenocarcinoma; MISP, Mitotic Spindle Positioning; GEPIA2, Gene Expression Profiling Interactive Analysis 2; GEO, Gene Expression Omnibus; HPA, Human Protein Atlas; TCGA, The Cancer Genome Atlas; FDR, false discovery rate; GO, Gene Ontology; KEGG, Kyoto Encyclopedia of Genes and Genomes; IPS, immunophenoscore; TMB, tumor mutational burden; TCIA, The Cancer Immunome Atlas; EMT, epithelial-mesenchymal transition; PAAD, pancreatic adenocarcinoma; IQGAP1, IQ motif containing GTPase activating protein 1; CP, chronic pancreatitis; CCK-8, Cell Counting Kit-8; ATCC, American Type Culture Collection; DEGs, differentially expressed genes; IC, immune checkpoint; ICI, immune checkpoint inhibitor; FC, fold change; IHC, immunohistochemistry; OS, overall survival; RFS, relapse-free survival; IRGs, immune-relevant genes; Tfh, follicular helper T cells; Tregs, regulatory T cells; CTLA-4, cytotoxic T-lymphocyte-associated protein 4; PD-1, programmed cell death protein 1; PD-L1, PD-1 ligand-1; PD-L2, PD-1 ligand-2; FAK, focal adhesion kinase.
