# Supplementary figures and images for: Up-Regulated MISP Is Associated With Poor Prognosis and Immune Infiltration in Pancreatic Ductal Adenocarcinoma
Source: Front Oncol. 2022 Mar 30;12:827051. doi: 10.3389/fonc.2022.827051 (PMC9005831; doi:10.3389/fonc.2022.827051)

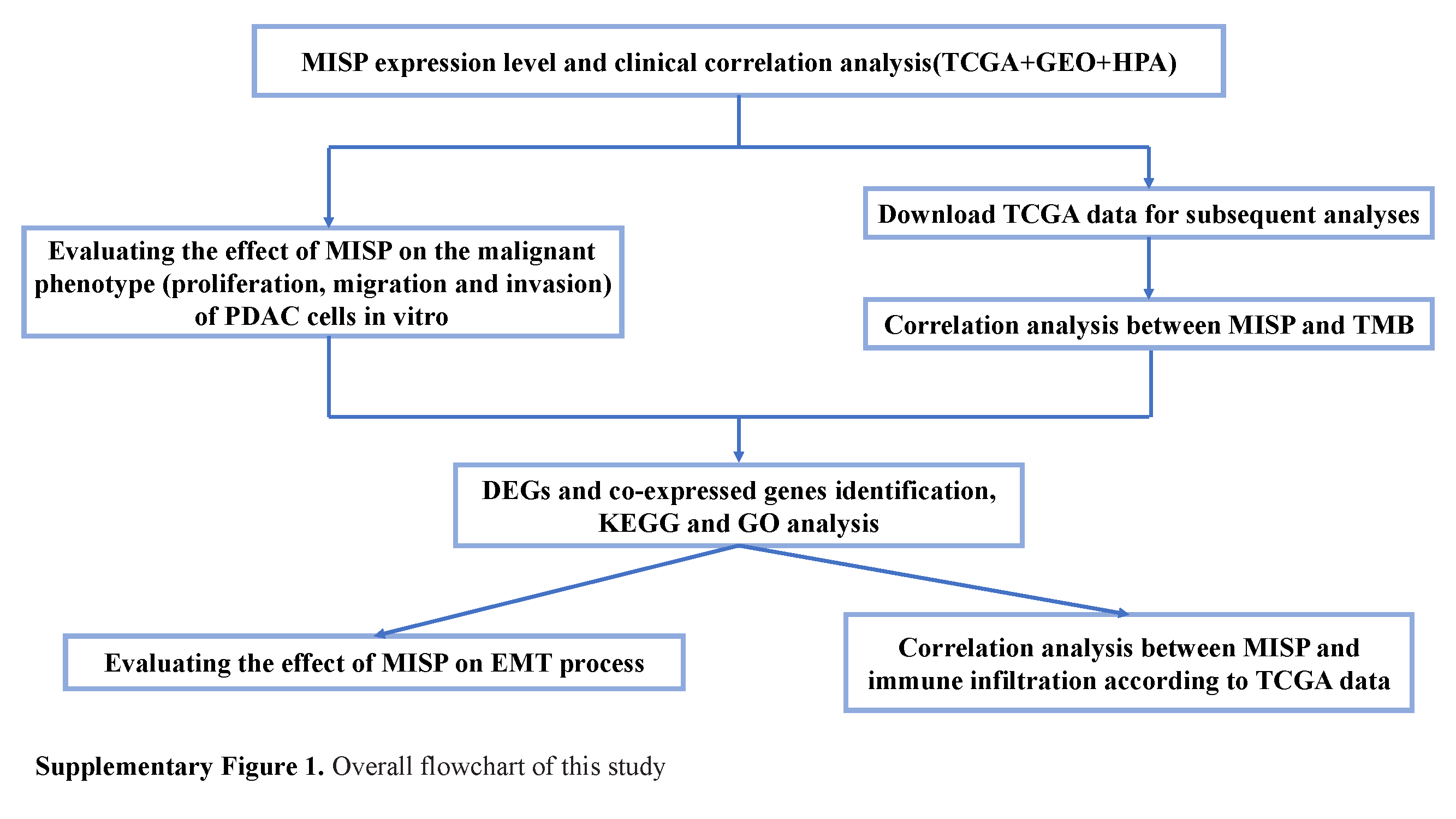

Supplement: Supplementary file 1 [file Image_1.tiff]

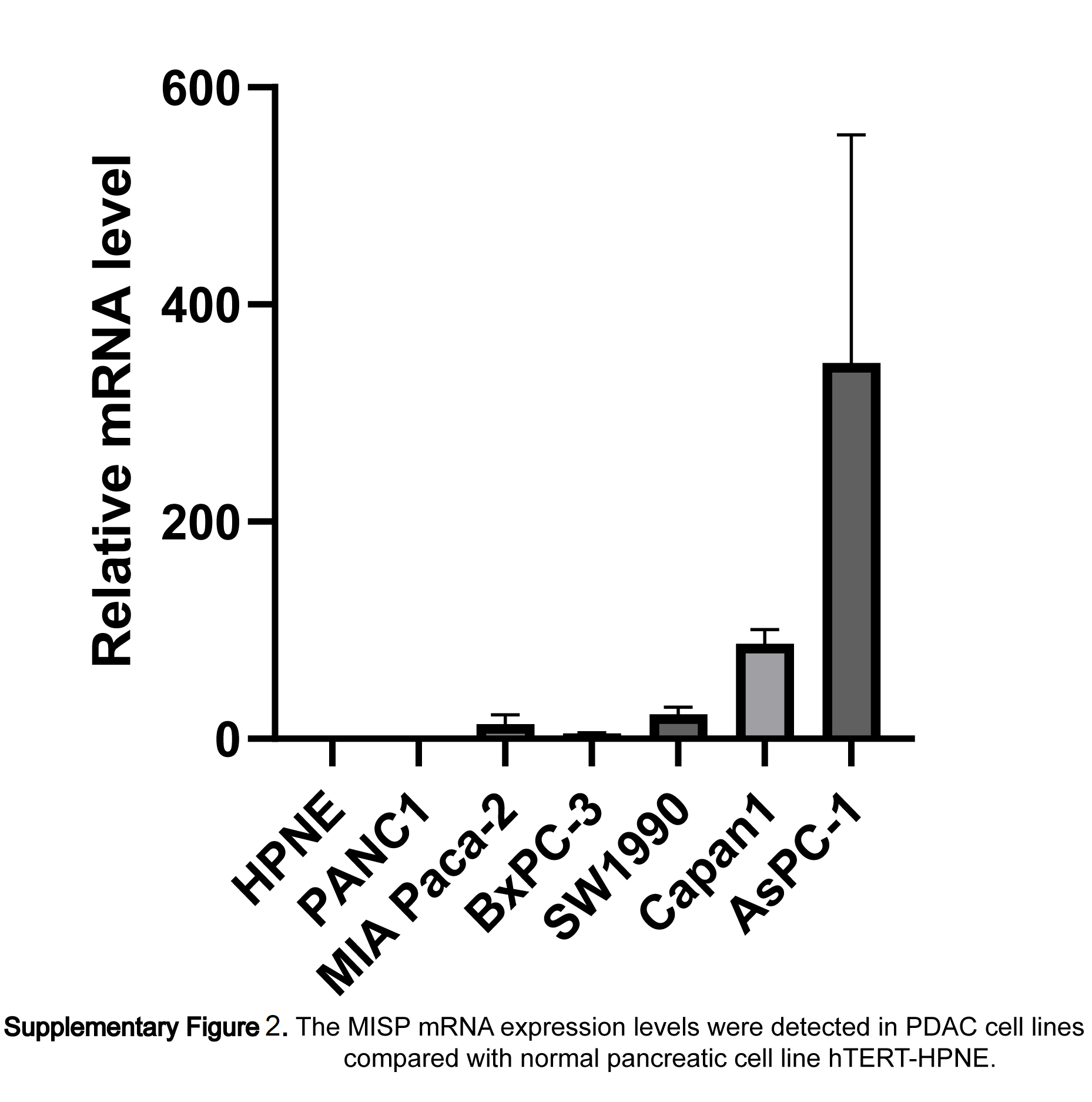

Supplement: Supplementary file 2 [file Image_2.tif]

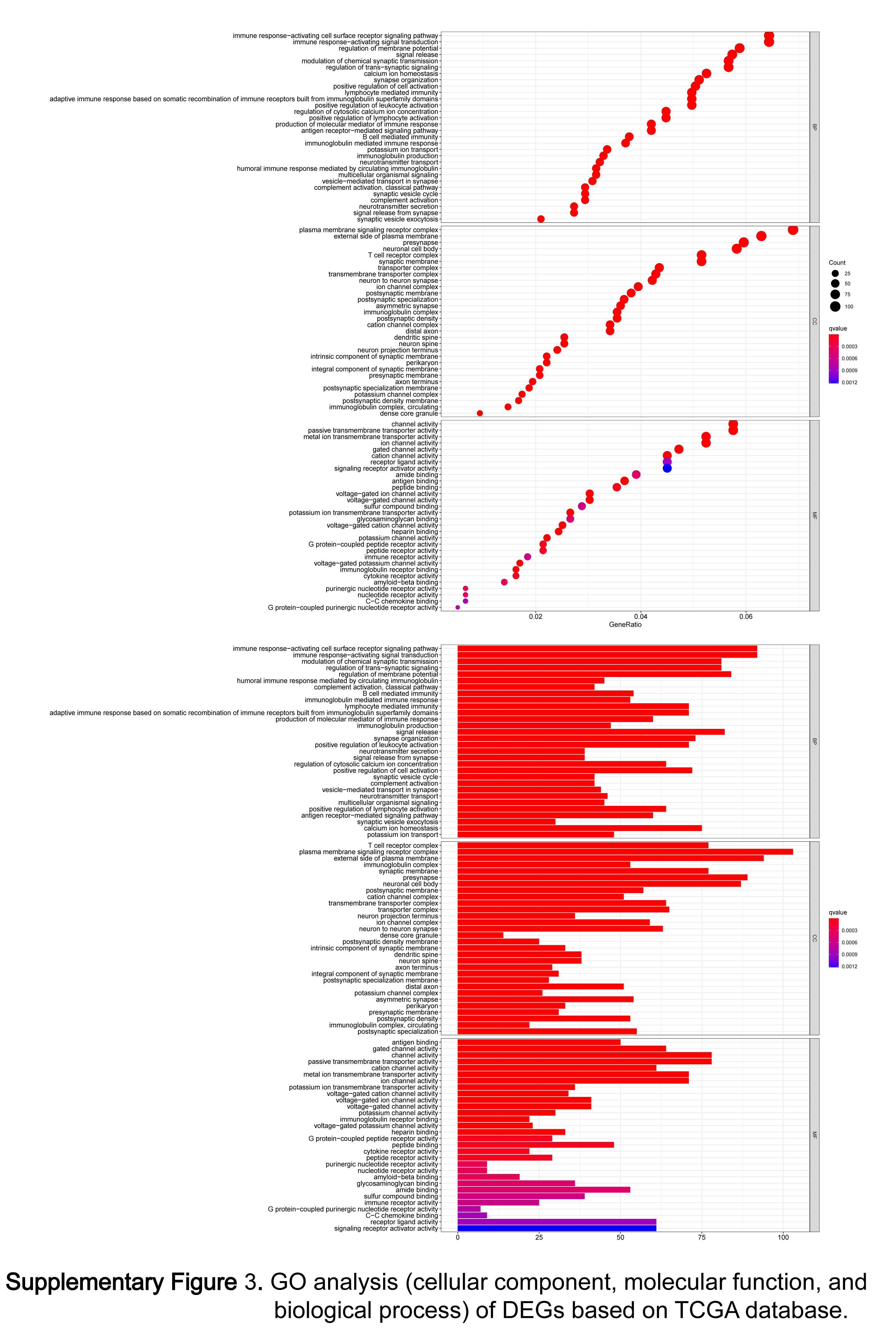

Supplement: Supplementary file 3 [file Image_3.tif]

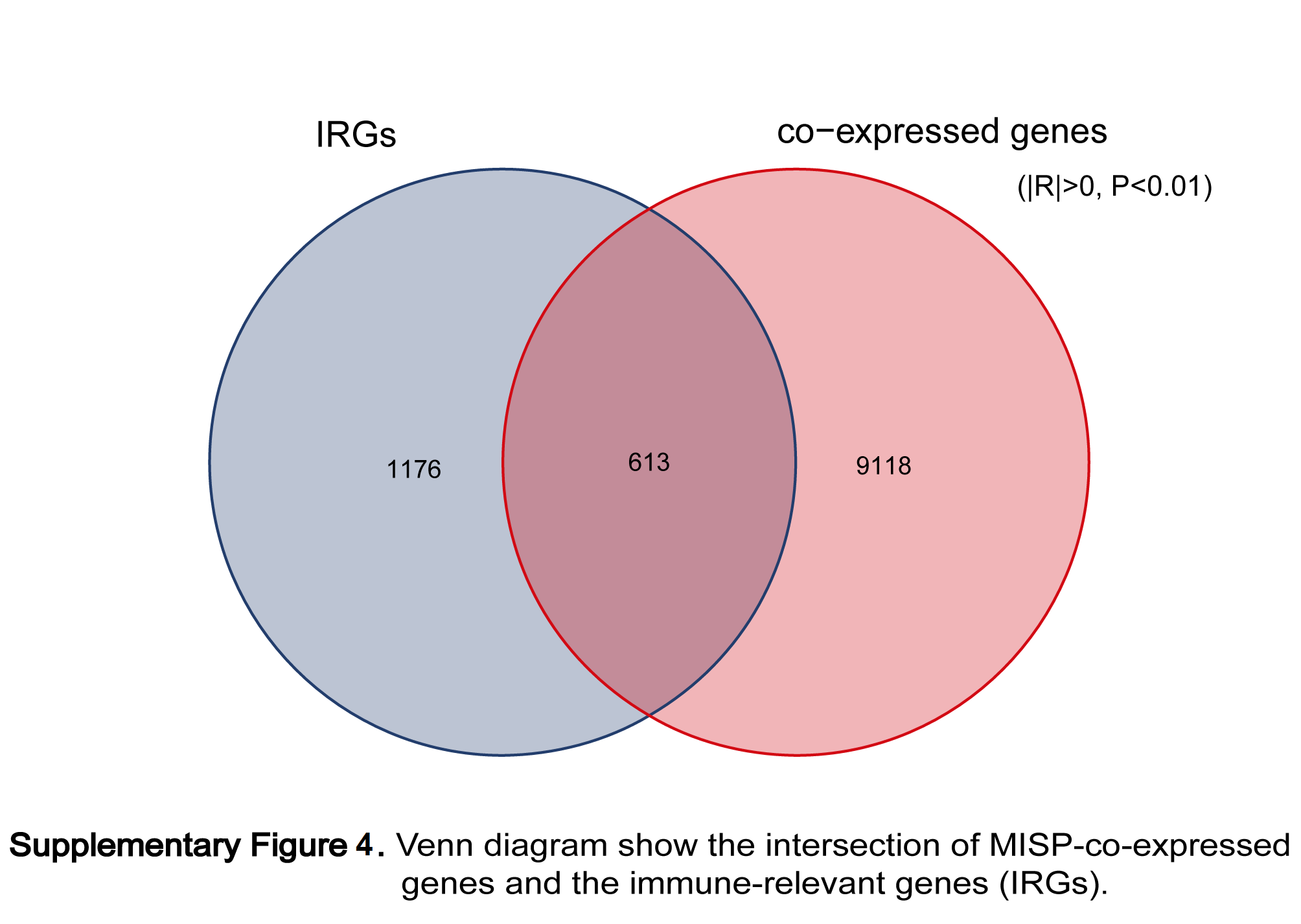

Supplement: Supplementary file 4 [file Image_4.tif]

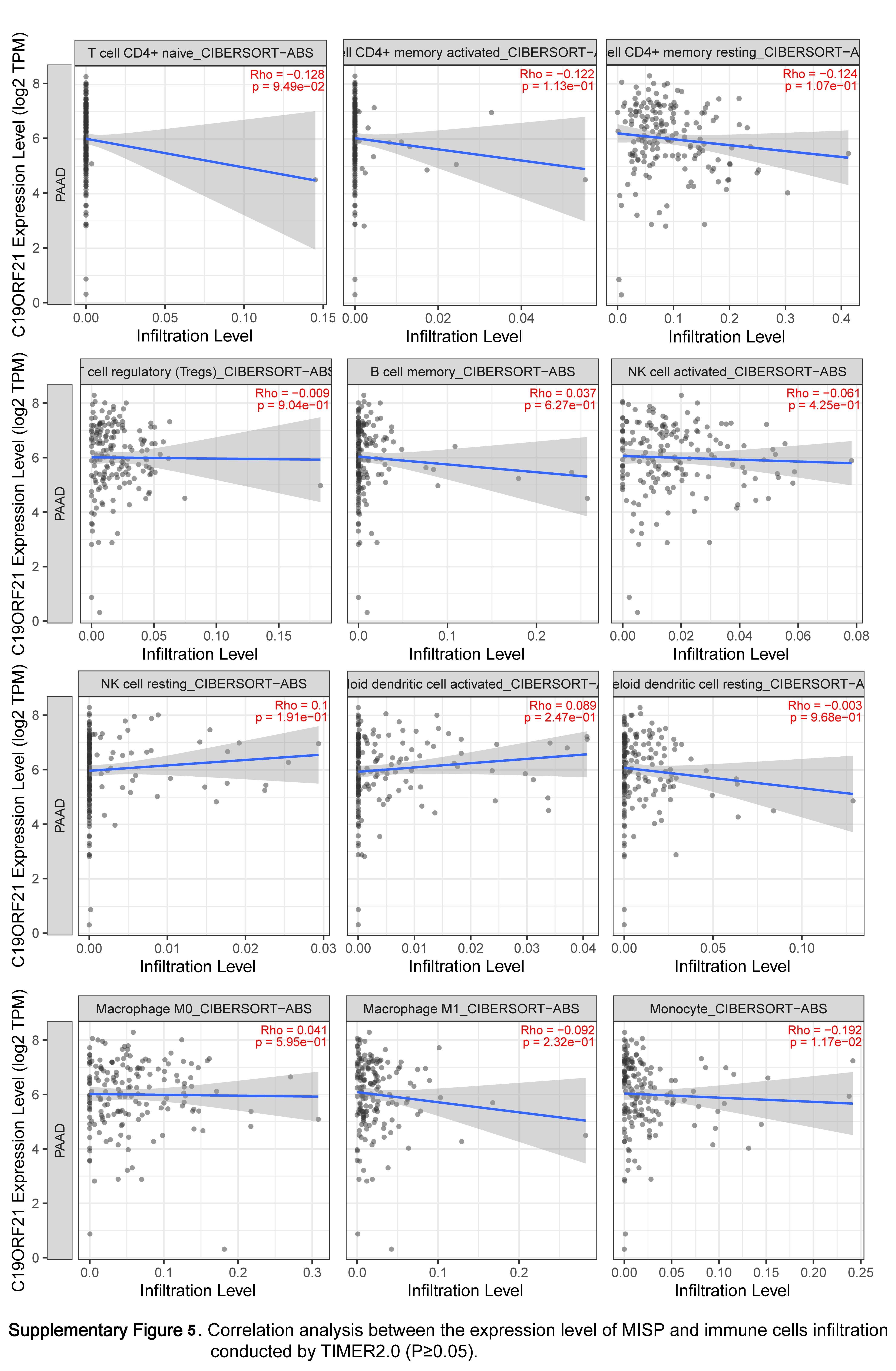

Supplement: Supplementary file 5 [file Image_5.tif]

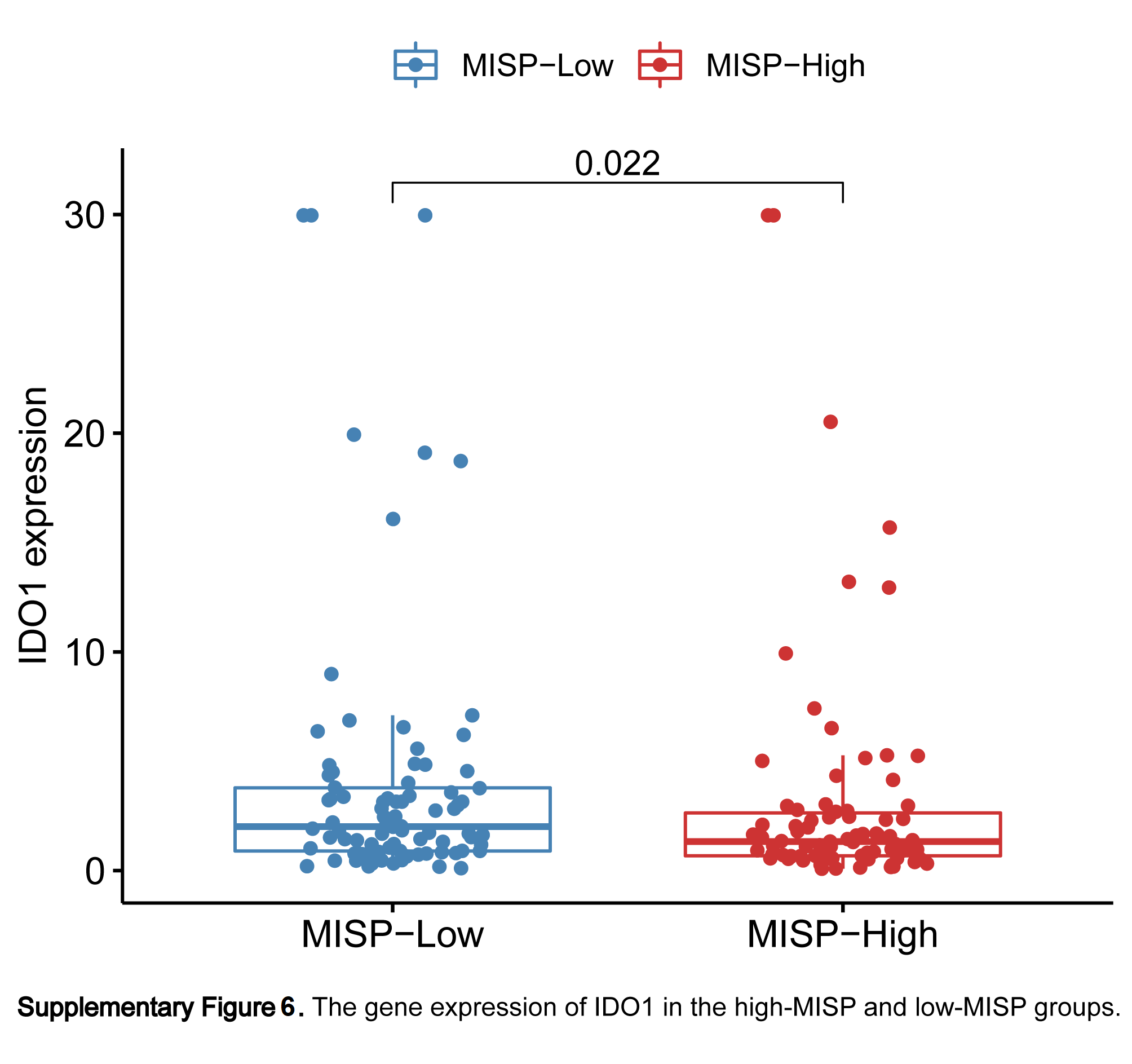

Supplement: Supplementary file 6 [file Image_6.tif]
